# Validation of the patient assessment of chronic illness care (PACIC) short form scale in heart transplant recipients: the international cross-sectional bright study

**DOI:** 10.1186/s12913-020-5003-3

**Published:** 2020-03-03

**Authors:** Katia Iglesias, Sabina De Geest, Lut Berben, Fabienne Dobbels, Kris Denhaerynk, L. Cynthia Russell, Remon Helmy, Isabelle Peytremann-Bridevaux, on behalf of the BRIGHT study team, Maria G. Crespo-Leiro, Paolo De Simone, Albert Groenewoud, Christiane Kugler, Linda Ohler, Johan Van Cleemput, Alain Jean Poncelet, Laurent Sebbag, Magali Michel, Andrée Bernard, Andreas Doesch, Ugolino Livi, Valentina Manfredini, Vicens Brossa-Loidi, Javier Segovia-Cubero, Luis Almenar-Bonet, Carmen Segura Saint-Gerons, Paul Mohacsi, Eva Horvath, Cheryl Riotto, Gareth Parry, Ashi Firouzi, Stella Kozuszko, Haissam Haddad, Annemarie Kaan, Grant Fisher, Tara Miller, Maureen Flattery, Kristin Ludrosky, Bernice Coleman, Jacqueline Trammell, Katherine St. Clair, Andrew Kao, Maria Molina, Karyn Ryan Canales, Samira Scalso de Almeida, Andrea Cotait Ayoub, Fernanda Barone, Michelle Harkess, Joanne Maddicks-Law

**Affiliations:** 1 School of Health Sciences (HEdS-FR), University of Applied Sciences and Arts of Western Switzerland (HES-SO), Route des Arsenaux 16a, 1700 Fribourg, Switzerland; 2 Institute of Nursing Science, Department Public Health, Faculty of Medicine, University of Basel, Bernoullistrasse 28, 4056 Basel, CH Switzerland; 3 Department of Public Health and Primary Care, Academic Centre for Nursing and Midwifery, KU Leuven, Kapucijnenvoer 35 blok d-box 7001, 3000 Leuven, Belgium; 4 School of Nursing and Health Studies, University of Missouri-Kansas City, Kansas City, MO USA; 5 Department of epidemiology and health systems (DESS), Center for Primary Care and Public Health (Unisanté), University of Lausanne, Route de la Corniche 10, 1010 Lausanne, Switzerland

**Keywords:** PACIC short form, Heart transplantation, Chronic care model, Language, Multi-center trial, Transplant team

## Abstract

**Background:**

Transplant recipients are chronically ill patients, who require lifelong follow-up to manage co-morbidities and prevent graft loss. This necessitates a system of care that is congruent with the Chronic Care Model. The eleven-item self-report Patient Assessment of Chronic Illness Care (PACIC) scale assesses whether chronic care is congruent with the Chronic Care Model, yet its validity for heart transplant patients has not been tested.

**Methods:**

We tested the validity of the English version of the PACIC, and compared the similarity of the internal structure of the PACIC across English-speaking countries (USA, Canada, Australia and United Kingdom) and across six languages (French, German, Dutch, Spanish, Italian and Portuguese). This was done using data from the cross-sectional international BRIGHT study that included 1378 heart transplant patients from eleven countries across 4 continents. To test the validity of the instrument, confirmatory factor analyses to check the expected unidimensional internal structure, and relations to other variables, were performed.

**Results:**

Main analyses confirmed the validity of the English PACIC version for heart transplant patients. Exploratory analyses across English-speaking countries and languages also confirmed the single factorial dimension, except in Italian and Spanish.

**Conclusion:**

This scale could help healthcare providers monitor level of chronic illness management and improve transplantation care.

**Trial registration:**

Clinicaltrials.gov ID: NCT01608477, first patient enrolled in March 2012, registered retrospectively: May 30, 2012.

## Background

Transplantation needs to be regarded as a chronic condition as patients remain dependent on life-long medical follow-up after transplantation and have to follow a complex therapeutic regimen. Moreover, new co-morbidities develop after transplantation, often due to the side effects of immunosuppressive drugs [1]. Worldwide, transplant recipients are thus part of a growing group of patients living with chronic diseases and multi-morbidity, representing a major burden for communities and healthcare systems in terms of morbidity, disability, mortality and healthcare costs.

Improving care for chronically ill patients implies a system of care that integrates building blocks of the Chronic Care Model [2, 3], i.e. support for self-management, delivery system design (e.g. continuity of care), decision support, clinical information systems, community resources and policies, as well as organization of healthcare. In fact, for more than a decade, the implementation of initiatives targeting better coordination and integration of care for patients with chronic disease based on the Chronic Care Model, has flourished [3–5]. Attention to care models in transplantation, based on principles of chronic illness management, has also increased [1, 6], and first evidence shows that reengineering transplant follow-up based on the principles of chronic illness management results in better adherence, clinical outcomes, better quality of life, and less health resource utilization [7, 8].

The Patient Assessment of Chronic Illness Care (PACIC) scale [9] assesses how chronic illness care, from the patient’s perspective, is congruent with the Chronic Care Model [2, 3]. It was developed by Glasgow et al. [9], and to our knowledge, has almost exclusively been used and validated in patient populations with highly prevalent chronic diseases such as diabetes [10–16], chronic pulmonary obstructive diseases [13], arthritis [17] and heart failure [16, 18]. Whereas these prevalent chronic diseases represent those with the highest societal burden in terms of morbidity, disability, mortality and cost [19–22], the level of chronic illness care in transplantation remains to be explored. In fact, transplant recipients represent a unique group of patients. They not only live with a chronic condition, as patients are not cured with a transplant, and often present with multiple co-morbidities requiring complex treatments and frequent follow-ups, but are also cared for in tertiary care facilities and always by multiple different healthcare professionals.

The PACIC could be used for assessing the level of chronic illness care in transplantation and provide insights in specific aspects of transplant management that could be improved. Moreover, the PACIC could also be used to benchmark transplant centers in relation to their level of chronic illness management, and this information can be taken into consideration when assessing variability in clinical outcomes among centers.

In this context, the primary goal of our study was to test the validity of the existing English version of the PACIC scale in a new group of patients, namely heart transplant recipients. The secondary – exploratory – goal of our study was to compare, in this population of chronic patients, the similarity of the internal structure of the PACIC across English-speaking countries and across various languages.

## Methods

### Study design

We performed secondary data-analyses, based on cross-sectional data from the BRIGHT study (Building Research Initiative Group: Chronic Illness Management and Adherence in Transplantation) [23], a multi-center, multi-continental study in heart transplantation aiming at describing chronic illness management practice patterns among centers, countries, and continents in heart transplantation. Detailed information on the BRIGHT study aims and methods can be found elsewhere [23].

Using a multi-stage sampling approach, a convenience sample of countries (i.e. Australia, Belgium, Brazil, Canada, France, Germany, Italy, Spain, Switzerland, the UK and the USA) and heart transplant centers (36 centers and minimally 2 per country) were included. Depending on the number of clinicians eligible, a random sample of a maximum of 5 clinicians per center were recruited in this study, and a random sample of 1677 among the 2523 eligible adult (> 18y) single-organ heart transplant recipients (1–5 years post-transplantation) were approached for participating (proportional sampling based on heart transplantation center volume). Details on inclusion/exclusion criteria and sample size calculation can be found in the BRIGHT methods paper [23].

### Sample

**Fig. 1:**
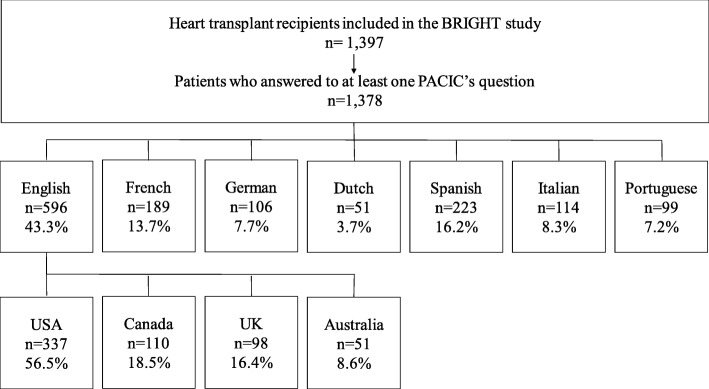
Distribution of the sample by language and by countries (within English-speaking countries)

Among the 1677 eligible participants, 244 declined and 36 died before completing the questionnaire, leaving 1397 participants. Out of the 1397 heart transplant recipients included in the BRIGHT study, 19 were excluded from our study population as they had not answered any of the PACIC questions/items. Among the 1378 patients who answered at least one PACIC question, 43% answered in English, 16% in Spanish, 13% in French, and less than 10% answered in Italian, in German, in Portuguese and in Dutch, respectively (for details see Fig. 1). Patients came from 36 centers which 11% are from non-urban areas (one Dutch-speaking center and three English-speaking centers) and 17% are from non-university teaching hospitals (three Portuguese-speaking centers and three English-speaking centers). There were 17 English-speaking centers, 5 Spanish-speaking centers, 6 French-speaking centers, four German-speaking centers, three Portuguese-speaking centers, three Italian-speaking centers and one Dutch-speaking center. There was one center with patients answering questionnaires in French, in German or in Italian (Switzerland), and another answering in English or in French (Canada).

The English population of patients (overall and by countries), which represents the main population of interest of the study, is described in Table 1. The mean age was 54.3 years, 70% of English speaking patients were men, and 52% of the causes underlying heart diseases were for idiopathic cardiomyopathy, 34% for ischemic cardiomyopathy, 4% congenital and 1% for valvular disease. Characteristics of the other language populations are presented in Appendix 1.

**Table 1:** Characteristics of English-speaking patients

	Whole English-speaking sample (*n* = 596)		USA (*n* = 337)		Canada (*n* = 110)		UK (*n* = 98)		Australia (*n* = 51)	
	(*n*)	mean (SD) / %	(*n*)	mean (SD) / %	(*n*)	mean (SD) / %	(*n*)	mean (SD) / %	(*n*)	mean (SD) / %
Age	(576)	54.3 (13.6)	(328)	56.2 (12.8)	(102)	54.8 (13.8)	(97)	49.1 (14.7)	(49)	50.6 (13.6)
Men (yes)	(591)	70.1	(334)	68.3	(109)	72.5	(97)	78.4	(51)	60.8
Caucasian (yes)	(586)	80.4	(333)	75.1	(108)	88.9	(98)	93.9	(47)	70.2
Civil status	(590)		(334)		(109)		(96)		(51)	
Single		19.3		17.4		15.6		27.1		25.5
Married/partnership		68.0		70.1		68.8		60.4		66.7
Divorced/separated/widowed		12.7		12.6		15.6		12.5		7.8
Education attainment	(592)		(336)		(108)		(98)		(50)	
Primary school		1.0		0.9		2.8		0.0		0.0
Secondary school		44.8		38.4		38.9		69.4		52.0
Higher education/University		54.2		60.7		58.3		30.6		48.0
Employment status (employed)	(582)	33.5	(329)	32.2	(108)	28.7	(98)	36.7	(47)	46.8
Causes of underling heart disease	(572)		(325)		(107)		(92)		(48)	
Congenital		4.4		1.2		4.7		15.2		4.2
Ischemic		33.7		40.9		28.0		19.6		25.0
Idiopathic		51.6		45.5		58.9		58.7		62.5
Valvular		1.1		1.2		0.0		1.1		2.1
Other		9.3		11.1		8.4		5.4		6.3
Charlson comorbidity	(596)	0.9	(337)	1.1	(110)	0.9	(98)	0.2	(51)	0.5
		(1.4)		(1.5)		(1.5)		(0.7)		(1.0)
Time post-transplantation (in years)	(585)	3.3 (1.4)	(333)	3.0 (1.3)	(105)	3.6 (1.5)	(98)	3.5 (1.2)	(49)	4.2 (1.5)

### Measures

The BRIGHT study used different sources of information (i.e. patient, clinician, heart transplant program director, medical file) to assess variables of interest, i.e. [1] the BRIGHT patient interview questionnaire, [4] the BRIGHT patient self-report (written) questionnaire, [1] the BRIGHT clinician questionnaire, [6] the BRIGHT transplant director questionnaire, and [7] the BRIGHT medical chart structured data collection form [23]. Following Wild et al. recommendations [24], patient questionnaires were translated from the original English version to Dutch, French, German, Italian, Spanish and Brazilian Portuguese by professional translators in a culturally sensitive way, and then pilot tested (further details are available elsewhere [23]). For this study, we will focus on the BRIGHT patient self-reported data.

To describe our sample, data included patients’ socio-demographics and health information: age (in year), gender (being male yes/no), ethnicity (Caucasian yes/no), education attainment (primary school, secondary school and college/university), employment status (being (self-)employed (part-time/full time paid work) yes/no), Charlson comorbidity index post heart transplantation [25] (19 items with weighted score, total ranging from 0 to 37), causes of underlying heart disease (congenital, ischemic, idiopathic, valvular, other) and number of year since transplantation.

To assess the level of chronic illness care from the patient’s perspective, we used the Patient Assessment of Chronic Illness Care (PACIC). More specifically we used the short version of the PACIC, an eleven-items self-reported measure [11, 18, 26, 27]. Patients were asked to score their care experience over the past 6 months in view of the eleven-items (for entire scale see footnote of Table 2) on a 5-point Likert scale: 1) none of the time, 2) a little of the time, 3) some of the time, 4) most of the time and 5) always.

**Table 2:** Distribution of the PACIC eleven-items: all English-speaking patients (*N* = 596)

				Response categories, %					
Item	Mean	(SD)	Median	Never	Generally not	Some-times	Most of the time	Always	Missing values
				1	2	3	4	5	
1	3.2	1.5	3	18.3	19.5	17.5	15.8	28.2	0.8
2	4.8	0.6	5	1.5	0.3	1.5	12.4	84.1	0.2
3	3.4	1.4	4	12.1	18.3	19.5	18.6	31.2	0.3
4	3.7	1.6	5	16.1	12.6	6.7	8.7	54.7	1.2
5	2.6	1.5	2	31.0	24.0	14.3	8.4	21.3	1.0
6	3.8	1.4	4	10.1	9.4	17.0	19.6	44.0	0.0
7	3.4	1.5	4	17.8	15.8	12.6	17.1	36.2	0.5
8	3.4	1.5	4	18.3	14.8	12.4	16.3	36.7	1.5
9	3.6	1.4	4	12.3	11.6	18.8	18.1	38.4	0.8
10	3.3	1.5	3	16.8	16.6	18.6	11.7	35.4	0.8
11	3.2	1.5	3	19.1	16.1	18.3	17.3	28.4	0.8

*SD* Standard deviation.

Items: 1) Given choices about treatment to think about; 2) Satisfied that my care was well organized. 3) Helped to set specific goals to improve my eating or exercise; 4) Given a copy of my treatment plan; 5) Encouraged to go to a specific group or class to help me cope with my heart transplantation; 6) Asked questions, either directly or with a questionnaire, about my health habits; 7) Helped to make a treatment plan that I could carry out in my daily life; 8) Helped to plan ahead so I could take care of my transplanted heart even in hard times; 9) Asked how my heart transplantation affects my life; 10) Contacted after a visit to see how things were going; 11) Told how my visits with other types of doctors, like an eye doctor or surgeon, helped my treatment [9]*.*

Finally, we also used questions relating to the transplant team: if the transplant team advised the patient to exercise during the past year (yes/no), if the transplant team discussed about the intake of immunosuppressive drugs in daily life (yes/no), the level of satisfaction with transplant team (mean of twelve questions going from 1 = very dissatisfied to 5 = very satisfied [28]) and the trust in the transplant team (mean of ten questions, going from 1 = strongly disagree’ (low trust) to ‘5= strongly agree’ (high trust) [29]).

### Validation process

Following the American Psychological Association guidelines of standards for educational and psychological testing, the validation process for an existing scale is composed by two steps: validation of the internal structure of the scale and the relation to other variables [30]. Actually, since we were not developing a new scale but working with the English version of an existing one, we did not repeat the content and response process steps (necessary to consider when validating a new scale) which had been conducted initially in English by Glasgow and his colleagues [9].

### Statistical analyses

First, we conducted descriptive analyses to characterize patients (percentage for ordinal or categorical data; means and standard deviation (SD) for continuous data) according to the language of the questionnaires they filled in, and, for English-speaking patients, according to their country. We also described the distribution of the PACIC eleven-items (mean, standard deviation, median, distribution by response category, and percentage of missing values) for each of these groups.

Second, we followed the first step of the American Psychological Association recommended validation process: validation of the internal structure of the scale. Therefore, we ran confirmatory factor analyses based on a polychoric correlation (correlation estimation between theorized normally distributed continuous latent variables measured through ordinal variables) matrix (weighted least squares estimation method (WLSMV) [31] to test the single dimension factorial structure of the PACIC [11, 18, 26, 27], and its expected internal structure. This type of confirmatory factor analyses was chosen because of the ordinal structure of the data (PACIC items measured on a 5-point Likert scale) [32]. Pairwise deletion technique (available-case analysis) was used with WLSMV to handle missing data.

Third, to explore the extent to which the internal structure was equivalent across English-speaking countries (USA, Canada, Australia and United Kingdom) and languages (French, German, Dutch, Spanish, Italian and Portuguese), we tested three forms of measurement invariance (i.e. measurement equivalence [33]): configural, metric and scalar invariance. This was done by comparing a series of multigroup confirmatory factor analyses gradually increasing model constraints. Whereas configural invariance requires the same factorial structure across groups, metric invariance requires equivalent loading across groups and scalar invariance requires equivalent thresholds across groups (for further details see [34–36]). In other words, configural invariance implies the same factorial structure across groups, metric variance implies that the loadings (the “weights” of each item) are similar across groups (i.e. the latent construct is understood similarly across groups) and scalar invariance allows conducting valid mean comparisons across groups. Goodness of fits of the various confirmatory factor analyses models were then tested using the Root Mean Square Error of Approximation (RMSEA) and Weighted Root Mean Square Residual (WRMR, recommended for ordinal data [37]); the latter two were checked jointly because of the sensitivity to misspecified factor loading for the RMSEA [38]. The Tucker Lewis index (TLI) and the Comparative Fit Index (CFI) were also presented since they are less affected by sample size [38, 39]. Models were considered to present a “good fit” if the RMSEA was < 0.05, [40], WRMR < 1.00 [37], TLI > 0.97 and CFI > 0.97 [41]. Models were considered to present an “acceptable fit” if RMSEA’s were between 0.05 and 0.08 and TLI’s and CFI’s between 0.95 and 0.97. For comparability reasons, we also computed ordinal alphas (i.e. ordinal reliability coefficients), instead of the usual Cronbach’s alpha because the latter takes into account the ordinal nature of the variables [42].

Finally, we conducted the second step of the standards for educational and psychological testing: the relation to other variables [30]. After having identified variables that we a priori hypothesized to be related to PACIC scores (i.e. variables related to the transplant team), we used them to explore associations between PACIC scores and the following variables: the transplant team’s advice for exercise during the past year (yes/no), discussion regarding intake of immunosuppressive with the transplant team, patient levels of satisfaction and levels of trust with the transplant team. Our hypotheses were that: a) patients who were advised to exercise will have higher PACIC scores than those who were not; b) patients who discussed intake of immunosuppressive medication with the transplant teams will have higher total PACIC scores than those who had not; c) high patients’ satisfaction with the transplant team will be correlated with high PACIC scores; and d) high levels of trust with the transplant team will be correlated with high PACIC scores. According to the different distributions of the latter variables, Spearman correlations were used to test the association between PACIC and continuous variables, whereas t-tests were used for independent groups in dummy variables. Analyses were conducted for the English version, English-speaking countries and different language groups. Whereas Stata 12 was used for most statistical analyses, Mplus 7 [31] was used to run confirmatory factor analyses and R [43] was used to run ordinal alphas.

## Results

### Level of chronic illness care from patient’s perspective (PACIC scores)

Table 2 provides the distribution of the PACIC eleven-items for all English-speaking patients. Whereas 96% of patients completed all items of the questionnaire and the percentage of missing values by item was very low (variation between 0 and 1.5%), a high percentage of patients ticked the highest response category (variation between 21 and 84%) with 8 out of eleven-items presenting ceiling effects (> 30% of the patients). On the other hand, the lowest response category was less often selected (variation between 1.5 and 19.1%), except for item 5 (31.0%), which can be considered as a floor effect. Details of the distribution of the PACIC items are shown in Appendixes 2a, 2b, 3a and 3b (across English-speaking countries and across language groups).

### PACIC structure

The results of confirmatory factor analyses based on a polychoric correlation matrix are presented in Tables 3 and 4, by countries for English-speaking patients, and by language groups for the other patients, respectively. Acceptable to good fits of the single factor structure (RMSEA < 0.08, WRMR <.1.00, CFI > 0.97, TLI > 0.97) were found for the English version of the PACIC and in all English-speaking countries except for one index for USA (RMSEA 0.092 instead of 0.080) and for Australia (RMSEA 0.087 instead of 0.080). Acceptable to good fits were found for the French and German versions. However, the Dutch version showed acceptable to good fits except for the RMSEA (0.110 instead of 0.080), and the Portuguese and the Italian versions showed at least two fits slightly away from the defined acceptable fits threshold. The Spanish version did not reach the expected thresholds.

**Table 3:** Loadings and model fits (confirmatory factor analyses based on polychoric correlation matrix) for the PACIC English version, overall and by countries

		Country			
Item	Whole English-speaking sample (*n* = 596)	USA (*n* = 337)	Canada (*n* = 110)	UK (*n* = 98)	Australia (*n* = 51)
1	0.65	0.63	0.57	0.82	0.61
2	0.39	0.45	0.49	0.29	0.34
3	0.82	0.80	0.81	0.84	0.81
4	0.76	0.66	0.74	0.78	0.78
5	0.78	0.77	0.72	0.81	0.87
6	0.74	0.64	0.74	0.79	0.90
7	0.90	0.85	0.89	0.88	0.96
8	0.91	0.87	0.89	0.95	0.96
9	0.79	0.77	0.84	0.76	0.94
10	0.78	0.69	0.74	0.83	0.83
11	0.78	0.76	0.72	0.77	0.70
*RMSEA*	*0.079*	*0.092*	*0.075*	*0.022*	*0.087*
*CFI*	*0.985*	*0.969*	*0.984*	*0.999*	*0.993*
*TLI*	*0.981*	*0.962*	*0.980*	*0.999*	*0.991*
*WRMR*	*0.985*	*0.987*	*0.600*	*0.478*	*0.531*
*Alpha ordinal*	*0.930*	*0.920*	*0.920*	*0.930*	*0.920*

*RMSEA* Root Mean Square Error of Approximation, *CFI* Comparative Fit Index, *TLI* The Tucker Lewis index, *WRMR* Weighted Root Mean Square Residual. Good Goodness of fit (GoF): RMSEA < 0.05, CFI > 0.97; TLI > 0.97; WRMR < 0.100; And acceptable GoF: RMSEA < 0.08, CFI > 0.95, TLI > 0.95.

**Table 4:** Loadings and model fits (confirmatory factor analyses based on polychoric correlation matrix) by language groups

Item	English (*n* = 596)	French (*n* = 189)	German (*n* = 106)	Dutch (*n* = 51)	Spanish (*n* = 223)	Italian (*n* = 114)	Portuguese (*n* = 99)
1	0.65	0.61	0.63	0.70	0.30	0.25	0.49
2	0.39	0.47	0.44	0.58	0.73	0.31	0.47
3	0.82	0.79	0.74	0.89	0.77	0.71	0.68
4	0.76	0.69	0.57	0.81	0.80	0.64	0.59
5	0.78	0.68	0.70	0.77	0.97	0.63	0.74
6	0.74	0.74	0.76	0.40	0.97	0.56	0.74
7	0.9	0.87	0.84	0.90	0.76	0.65	0.84
8	0.91	0.95	0.87	0.90	0.81	0.84	0.83
9	0.79	0.81	0.74	0.70	0.75	0.71	0.67
10	0.78	0.76	0.69	0.62	0.76	0.48	0.69
11	0.78	0.58	0.71	0.77	0.80	0.68	0.60
*RMSEA*	*0.079*	*0.074*	*0.070*	*0.110*	*0.169*	*0.075*	*0.087*
*CFI*	*0.985*	*0.984*	*0.982*	*0.971*	*0.970*	*0.948*	*0.947*
*TLI*	*0.981*	*0.980*	*0.977*	*0.964*	*0.962*	*0.934*	*0.933*
*WRMR*	*0.985*	*0.719*	*0.625*	*0.664*	*1.879*	*0.778*	*0.719*
*Alpha ordinal*	*0.930*	*0.920*	*0.900*	*0.890*	*0.900*	*0.810*	*0.880*

*RMSEA* Root Mean Square Error of Approximation, *CFI* Comparative Fit Index, *TLI* The Tucker Lewis index, *WRMR* Weighted Root Mean Square Residual. Good Goodness of fit (GoF): RMSEA < 0.05, CFI > 0.97; TLI > 0.97; WRMR < 1.00; And acceptable GoF: RMSEA < 0.08, CFI > 0.95, TLI > 0.95.

Loadings were similar both across English-speaking countries and between the English, French and German versions. The Dutch and the Portuguese versions did not differ too much for the latter and appeared to be substantially different for the Spanish and the Italian versions, particularly for items 1 and 2.

Configural invariance was established across English-speaking countries, meaning that the factorial structure was identical (i.e. a single dimension) across English-speaking countries. This was not the case for the other language groups. Indeed, whereas the Spanish version did not show acceptable fits, the Spanish and the Italian patients did not use the whole scale of answering, i.e. some anchors (response modality) for item 2 and 4 were never chosen, which prevented us from testing multigroup invariance. Despite these results, we found configural invariance between the English, French, German, Dutch and Portuguese versions with acceptable fits (RMSEA =0.073, CFI =0.986, TLI =0.982). Metric invariance was found neither across English-speaking countries nor across language groups.

### Relationship between PACIC’s score and variables of the field

For the English version, the hypothesized relationships between PACIC scores and the variables related to transplant team were confirmed (Table 5). In fact, we found that: a) patients advised to exercise reported higher PACIC scores than those who were not; b) patients with whom immunosuppressive medication intake had been discussed reported higher PACIC scores than those with whom it had not; c) higher levels of satisfaction with the transplant team were associated with higher PACIC scores; and d) higher levels of trust were correlated with higher PACIC scores. Similar results were found across English speaking countries (for details, see appendix 4), except for the UK, where no significant differences were found between the PACIC scores of patients who had received advice to exercise and those who had not, and no significant relation was identified between the PACIC scores and the levels of trust in the transplant team.

**Table 5:** relations between PACIC global score and other variables by languages

	English (*n* = 596)		French (*n* = 189)		German (*n* = 106)		Dutch (*n* = 51)		Spanish (*n* = 223)		Italian (*n* = 114)		Portuguese (*n* = 99)	
Advice to exercise (yes = 1, no = 0) (1)	1.0	***	0.7	**	0.7	**					0.1	ns		
Transplant team discussed intake immunosuppressants (yes = 1, no = 0) (1)	1.1	***							1.0	***				
Satisfaction with transplant team (2)	0.364	***	0.216	**	0.439	***	0.271	.	0.375	***	0.323	***	0.058	ns
Trust in transplant team (2)	0.268	***	0.293	***	0.400	***	0.128	ns	0.324	***	0.211	*	−0.012	ns

Statistical test: (1) t-test: mean differences, (2) spearman correlation; *p*-value: *** *p* < 0.001; ** *p* < 0.01; * *p* < 0.05; *p* < 0.10; ns non-significant *p*-value; N.B.: empty cells when category “no” less than 10 persons.

Our hypotheses were also confirmed for the French, German and Spanish versions, whereas they were only partially confirmed for the Italian version of the questionnaire (no significant differences were found between the PACIC scores of patients who received advice to exercise and those who did not), and were not confirmed at all for the Dutch and Portuguese versions.

## Discussion

This study aimed to test the validity of the existing English version of the PACIC scale for heart transplant recipients, a new group of chronically ill patients not considered in previous PACIC studies. As a result, the examination of the internal structure of the scale showed goodness of fit values within acceptable range for a single dimension, and the analysis of the relationships with the variables tested confirmed our hypotheses. In fact, perceived level of chronic illness management (PACIC scores) appeared to be positively associated with treatment satisfaction and levels of trust in the transplantation team, and higher perceived levels of chronic illness management were identified when patients had been advised to exercise and when the immunosuppressive treatment had been discussed. This study also intended to explore the extent to which the internal structure of the questionnaire was equivalent in different English-speaking countries (USA, Canada, Australia and United Kingdom) and across language groups (French, German, Dutch, Spanish, Italian and Portuguese). We confirmed the unique factorial dimension across English-speaking countries as well as for the French, German, Dutch and Portuguese versions of the questionnaire but not for the Italian and the Spanish versions of the scale. In addition, while hypothesized relationships between perceived level of chronic illness management and other variables were coherent across English-speaking countries and for the French, German and Spanish versions of the questionnaire, respectively, they were not for the remaining languages (i.e. Dutch, Italian and Portuguese).

Confirming the structural dimension of a questionnaire (i.e. configural invariance, one dimension in the case of the PACIC) is necessary but not sufficient to compare scores across groups and languages. With configural invariance the latent factor of the questionnaire is composed by the same items but this does not mean that they are included in the same proportion (metric invariance) or that the anchors (response modalities) are identical across groups and between languages (scalar invariance). Our analyses did not confirm metric invariance criteria: even if the items composing the PACIC score were the same, they were not combined similarly across groups and languages. This suggests that patients speaking different languages do not interpret/understand the questionnaire similarly. In fact, to ensure a common understanding of self-perceived integrated care, the PACIC score – the latent factor of the PACIC scale - should be composed of at least the same proportion of each item across English speaking countries and across languages (i.e. metric invariance). Varying meanings or interpretations can arise from cultural or/and healthcare system differences indeed. The latter two phenomena, which cannot easily be disentangled, represent key explanations of existing differences to consider when making international comparisons. We nevertheless have to remember that questionnaires (e.g. PACIC) are most often developed for clinical purposes such as following the evolution of a patient state (e.g. comparison of patients’ scores over time) or investigating the impact of an intervention (e.g. comparison of patients’ scores before and after an intervention). This means that any international comparisons should be made with great caution, given that those results represent a detour of the questionnaires’ initial use. Currently, further explorations of the impact of cultural and healthcare system differences on the assessment of provided care are needed.

As mentioned before, we found a positive association between the PACIC score and satisfaction results. Among the twelve satisfaction items, two (‘how consistent the information of the team was’ and ‘how understandable their information was’) are also part of the Chronic Care Model measured with the PACIC. This may contribute to the positive correlation between satisfaction and the perceived level of chronic illness management. The other satisfaction items focus on how the transplantation team takes care of patients by listening and being present (and also, for example, by being ‘friendly’, ‘encouraging’ and ‘supportive’). These communication items probably allow the patient to ask questions and to obtain information on his/her disease. As such, patients may be reassured and report better chronic illness management. This is also supported by the positive association found between trust in the transplantation team and level of chronic illness management. In fact, trust in the transplantation team and care satisfaction should remain central when considering the integration of care of chronically ill patients.

This study presents two main strengths. Firstly, we targeted a population of chronically ill patients which were not previously involved in PACIC-related studies. Secondly, we used data from patients from a variety of countries and languages, allowing us to obtain a more comprehensive perspective on the validity of the PACIC scale. Nevertheless, two limitations need to be addressed. First, although the overall sample is large, the sample size per language or country was low for Dutch, Portuguese, UK and Australia (less than 100) and German, French, Italian and Canada (less than 200), which may explain why acceptable fits were not always reached. In fact, Muthén and Muthén [44] suggest a minimum of 300 patients when working with ordinal data; a number that was only reached when pooling the English-speaking countries or for the USA. A second limiting factor lies in the fact that some language groups included patients from countries with different cultures and health care systems. Indeed, pooling countries speaking and using the same language does not mean that the same context/culture is shared. For example, differences in healthcare systems or expectations as well as care experiences may influence the way PACIC items are understood by patients. These previous reasons brought us to consider our secondary aim - similarity of the internal structure of the PACIC across English-speaking countries and across various languages - as only exploratory.

## Conclusion

Heart transplant patients represent an increasing group of chronically ill patients requiring complex treatments as well as life-long follow-up in a variety of care structures and with multiple healthcare professionals. Our results show that the English version of the PACIC, a scale assessing how level of chronic illness care perceived by patients is consistent with the recommendations of the Chronic Care Model, is valid in an international sample of heart transplant patients. Further validation efforts are needed since this scale is also very likely to be relevant for other solid organ transplant groups of patients who are also chronically ill. In the field of transplantation care, this tool could help healthcare professionals and providers in monitoring chronic illness management over time, adjusting it to patients’ needs and experiences, and thus improving transplantation care as well as providing insights into specific aspects of transplant management that could be improved. While awaiting results of further research comparing patients over time or comparing groups of patients where only the type of intervention varies, comparisons across countries or languages should be performed with caution since interpretation of the questions by patients can differ depending on the context, culture or healthcare system.

## Supplementary information

**Additional file 1: Appendix 1.** Characteristics of patients, by PACIC language. **Appendix 2a.** Distribution of the 11 PACIC items, by English-speaking countries (%)..**Appendix 2b.** Distribution of the 11 PACIC items, by English-speaking countries (%). **Appendix 3a.** Distribution of the 11 PACIC items by language groups (%). **Appendix 3b.** Distribution of the 11 PACIC items by language groups (%). **Appendix 4.** Relations between PACIC global score and other variables by English speaking.

## Data Availability

The datasets generated and/or analyzed during the current study are not publicly available as we have committed to confidentiality to the 36 participating centers at start of the BRIGHT project in 2012. A data sharing protocol has been agreed upon among all 36 centers of the BRIGHT project for internal use.

## Abbreviations

BRIGHT: Building Research Initiative Group: Chronic Illness Management and Adherence in Transplantation
CFI: Comparative Fit Index
PACIC: Patient Assessment of Chronic Illness Care
RMSEA: Root Mean Square Error of Approximation
TLI: The Tucker Lewis index
WLSMV: Weighted least squares estimation method
WRMR: Weighted Root Mean Square Residual

## References

[1] De Geest SM, Dobbels F, Gordon E, De Simone P (2011). Chronic illness management as an innovative pathway for enhancing long-term survival in transplantation. Am J Transplant.

[2] Wagner E, Austin B, Davis C, Hindmarsh M, Schaefer J, Bonomi A (2001). Improving chronic illness care: translating evidence into action. Health Aff Millwood.

[3] Epping-Jordan J, Pruitt S, Bengoa R, Wagner E (2004). Improving the quality of health care for chronic conditions. Qual Saf Health Care.

[4] Büsse R, Blümel M, Scheller-Kreinsen D, Zentner A (2010). Tackling Chronic Disease In Europe. Strategies, interventions and challenges.

[5] Nuño R, Coleman K, Bengoa R, Sauto R (2012). Integrated care for chronic conditions: the contribution of the ICCC framework. Health Policy.

[6] De Geest SM, Denhaerynck K, Berben L, Vanhaecke J, Russell C, Dobbels F (2015). Higher level of chronic illness Management in Heart Transplant Centers is associated with better patient survival: the intercontinental BRIGHT study. Circulation..

[7] Bissonnette JM, Woodend K, Davies BL, Stacey D, Knoll GA (2013). Evaluation of a collaborative chronic care approach to improve outcomes in kidney transplant recipients. Clin Transpl.

[8] Schmid A, Hils S, Kramer-Zucker A, Bogatyreva L, Hauschke D, De Geest SM, et al. Telemedically Supported Case Management for Living Donor Renal Transplant Recipients as Optimization Tool of Routine Evidence-Based Aftercare: A Single-Center Randomized Controlled Trial. Am J Transplant. 2017 [cited 2017 Mar 18];in press. Available from. 10.1111/ajt.14138. 10.1111/ajt.14138 27873477

[9] Glasgow R, Wagner E, Schaefer J, Mahoney L, Reid R, Green S (2005). Development and validation of the patient assessment of chronic illness care. Med Care.

[10] Aragones A, Schaefer E, Stevens D, Gourevitch M, Glasgow R, Shah N (2008). Validation of the Spanish translation of the patient assessment of chronic illness care (PACIC) survey. Prev Chronic Dis.

[11] Gugiu PC, Coryn C, Clark R, Kuehn A (2009). Development and evaluation of the short version of the patient assessment of chronic illness care instrument. Chronic Illn.

[12] Gugiu C, Coryn C, Applegate B (2010). Structure and measurement properties of the patient assessment of chronic illness care instrument. J Eval Clin Pract.

[13] Wensing M, Van Lieshout J, Jung H, Hermsen J, Rosemann T (2008). The patient assessment chronic illness care (PACIC) questionnaire in the Netherlands: a validation study in rural general practice. BMC Health Serv Res.

[14] Drewes H, de Jong-van Til J, Struijs J, Baan C, Tekle F, Meijboom B (2013). Measuring chronic care management experience of patients with diabetes: PACIC and PACIC+ validation. Int J Integr Care.

[15] Maindal H, Sokolowski I, Vedsted P (2010). Adaptation, data quality and confirmatory factor analysis of the Danish version of the PACIC questionnaire. Eur J Pub Health.

[16] Taggart J, Chan B, Jayasinghe U, Christl B, Proudfoot J, Crookes P (2011). Patients assessment of chronic illness care (PACIC) in two Australian studies: structure and utility. J Eval Clin Pract.

[17] Rosemann T, Laux G, Droesemeyer S, Gensichen J, Szecsenyi J (2007). Evaluation of a culturally adapted German version of the patient assessment of chronic illness care (PACIC 5A) questionnaire in a sample of osteoarthritis patients. J Eval Clin Pract.

[18] Cramm JM, Nieboer AP (2012). The chronic care model: congruency and predictors among patients with cardiovascular diseases and chronic obstructive pulmonary disease in the Netherlands. BMC Health Serv Res.

[19] World Health Organization. Global Health Estimates. Deaths by cause, age, sex, by country and by region, 2000-2015. Geneva; 2015. 2016. Available from: http://www.who.int/healthinfo/global_burden_disease/estimates/en/index1.html.

[20] GBD 2015 Disease and Injury Incidence and Prevalence Collaborators (2016). Global, regional, and national incidence, prevalence, and years lived with disability for 310 diseases and injuries, 1990–2015: a systematic analysis for the Global Burden of Disease Study 2015. Lancet.

[21] GBD 2015 Mortality and Causes of Death Collaborators (2016). Global, regional, and national life expectancy, all-cause mortality, and cause-specific mortality for 249 causes of death, 1980–2015: a systematic analysis for the Global Burden of Disease Study 2015. Lancet.

[22] Muka T, Imo D, Jaspers L, Colpani V, Chaker L, van der Lee SJ (2015). The global impact of non-communicable diseases on healthcare spending and national income: a systematic review. Eur J Epidemiol.

[23] Berben L, Denhaerynck K, Dobbels F, Engberg S, Vanhaecke J, Crespo-Leiro MG (2015). Building research initiative group: chronic illness management and adherence in transplantation (BRIGHT) study: study protocol. J Adv Nurs.

[24] Wild D, Grove A, Martin M, Eremenco S, McElroy S, Verjee-Lorenz A (2005). Principles of good practice for the translation and cultural adaptation process for patient-reported outcomes (PRO) measures: report of the ISPOR task force for translation and cultural adaptation. Value Health.

[25] Charlson ME, Pompei P, Ales KL, MacKenzie CR (1987). A new method of classifying prognostic comorbidity in longitudinal studies: development and validation. J Chronic Dis.

[26] Goetz K, Freund T, Gensichen J, Miksch A, Szecsenyi J, Steinhaeuser J (2012). Adaptation and psychometric properties of the PACIC short form. Am J Manag Care.

[27] Iglesias K, Burnand B, Peytremann-Bridevaux I (2014). PACIC instrument: disentangling dimensions using published validation models. Int J Qual Health Care.

[28] Westaway MS, Rheeder P, Van Zyl DG, Seager JR (2003). Interpersonal and organizational dimensions of patient satisfaction: the moderating effects of health status. Int J Qual Health Care.

[29] Hall MA (2006). Researching medical trust in the United States. J Health Organ Manag.

[30] American Educational Research Association (2014). American Psychological Association., National Council on Measurement in Education., & Joint Committee on Standards for Educational and Psychological Testing (U.S.). Standards for educational and psychological testing.

[31] Muthén L, Muthén B (2010). Mplus user’s guide.

[32] Muthén BO (1984). A general structural equation model with dichotomous, ordered categorical, and continuous latent variable indicators. Psychometrika..

[33] Byrne BM, Shavelson RJ, Muthén B (1989). Testing for the equivalence of factor covariance and mean structures: the issue of partial measurement invariance. Psychol Bull.

[34] Byrne BM (2012). Structural equation modeling with Mplus: basic concepts, applications, and programming.

[35] Vandenberg RJ, Lance CE (2000). A review and synthesis of the measurement invariance literature: suggestions, practices, and recommendations for organizational research. Organ Res Methods.

[36] Gregorich SE (2006). Do self-report instruments allow meaningful comparisons across diverse population groups? Testing measurement invariance using the confirmatory factor analysis framework. Med Care.

[37] Yu CY, Muthén B (2002). Evaluation of model fit indices for latent variable models with categorical and continuous outcomes.

[38] Hu L, Bentler PM (1998). Fit indices in covariance structure modeling: sensitivity to underparameterized model misspecification. Psychol Methods.

[39] Marsh RPHW, Balla JR, McDonald (1988). Goodness-of-fit indexes in confirmatory factor analysis: The effect of sample size. Psychol Bull.

[40] Browne MWCR, Bolen KALJS (1993). Testing structural equation models. Newbury.

[41] Schermelleh-Engel K, Moosbrugger H, Müller H (2003). Evaluating the fit of structural equation models: tests of significance and descriptive goodness-of-fit measures. Methods Psychol Res Online.

[42] Zumbo BD, Gadermann AM, Zeisser C (2007). Ordinal versions of coefficients alpha and Theta for Likert rating scales. J Mod Appl Stat Methods.

[43] Core R, Team R (2013). a language and environment for statistical computing.

[44] Muthén LK, Muthén BO (2002). How to use a Monte Carlo study to decide on sample size and determine power. Struct Equ Model Multidiscip J.

