# Supplementary material for: Validation of the patient assessment of chronic illness care (PACIC) short form scale in heart transplant recipients: the international cross-sectional bright study
Source: BMC Health Serv Res. 2020 Mar 3;20:160. doi: 10.1186/s12913-020-5003-3 (PMC7055084; doi:10.1186/s12913-020-5003-3)
Supplement: Supplementary file 1 — Additional file 1: Appendix 1. Characteristics of patients, by PACIC language. Appendix 2a. Distribution of the 11 PACIC items, by English-speaking countries (%)..Appendix 2b. Distribution of the 11 PACIC items, by English-speaking countries (%). Appendix 3a. Distribution of the 11 PACIC items by language groups (%). Appendix 3b. Distribution of the 11 PACIC items by language groups (%). Appendix 4. Relations between PACIC global score and other variables by English speaking. [file 12913_2020_5003_MOESM1_ESM.docx]

**Appendix 1: Characteristics of patients, by PACIC language**

|  | French (n=189) | | German (n=106) | | Dutch (n=51) | | Spanish (n=223) | | Italian (n=114) | | Portuguese (n=99) | |
| --- | --- | --- | --- | --- | --- | --- | --- | --- | --- | --- | --- | --- |
|  | **(n)** | mean (SD) / % | **(n)** | mean (SD) / % | **(n)** | mean (SD) / % | **(n)** | mean (SD) / % | **(n)** | mean (SD) / % | **(n)** | mean (SD) / % |
| Age | (185) | 51.1 (12.8) | (105) | 53.1 (12.6) | (51) | 52.1 (13) | (220) | 56.3 (11.8) | (110) | 57 (12.6) | (98) | 47 (13.2) |
| Gender male (yes) | (189) | 72.5 | (105) | 74.3 | (51) | 72.6 | (223) | 76.2 | (114) | 83.3 | (99) | 67.7 |
| Caucasian (yes) | (188) | 91.0 | (105) | 96.2 | (51) | 100.0 | (220) | 91.4 | (114) | 99.1 | (99) | 64.7 |
| Civil status | (188) |  | (105) |  | (51) |  | (223) |  | (113) |  | (99) |  |
| Single |  | 22.3 |  | 14.3 |  | 5.9 |  | 11.7 |  | 12.4 |  | 24.2 |
| Married/partnership |  | 63.8 |  | 73.3 |  | 84.3 |  | 69.5 |  | 75.2 |  | 64.7 |
| Divorced/separated/widowed |  | 13.8 |  | 12.4 |  | 9.8 |  | 18.8 |  | 12.4 |  | 11.1 |
| Education attainment | (187) |  | (105) |  | (50) |  | (216) |  | (114) |  | (96) |  |
| Primary school |  | 7.0 |  | 9.5 |  | 2.0 |  | 43.1 |  | 32.5 |  | 26.0 |
| Secondary school |  | 74.9 |  | 72.4 |  | 78.0 |  | 38.9 |  | 49.1 |  | 53.1 |
| Higher education/University |  | 18.2 |  | 18.1 |  | 20.0 |  | 18.1 |  | 18.4 |  | 20.8 |
| Employment status (employed) | (185) | 32.5 | (104) | 24.1 | (50) | 30.0 | (220) | 8.2 | (114) | 27.2 | (99) | 18.2 |
| Causes of underling heart disease | (188) |  | (104) |  | (51) |  | (222) |  | (114) |  | (92) |  |
| Congenital |  | 2.7 |  | 2.9 |  | 3.9 |  | 2.7 |  | 2.6 |  | 1.1 |
| Ischemic |  | 30.9 |  | 38.5 |  | 41.2 |  | 30.6 |  | 35.1 |  | 17.4 |
| Idiopathic |  | 50.5 |  | 46.2 |  | 49.0 |  | 56.3 |  | 56.1 |  | 34.8 |
| Valvular |  | 6.4 |  | 2.9 |  | 2.0 |  | 6.8 |  | 2.6 |  | 4.4 |
| Other |  | 9.6 |  | 9.6 |  | 3.9 |  | 3.6 |  | 3.5 |  | 42.4 |
| Charlson comorbidity index | (189) | 1.0 (1.3) | (106) | 1.7  (1.5) | (51) | 0.9  (1.2) | (223) | 0.9  (1.3) | (114) | 1.6  (1.3) | (99) | 0.9  (1.5) |
| Time post-transplantation (in years) | (188) | 3.7 (1.4) | (106) | 3.4 (1.3) | (51) | 3.4 (1.2) | (220) | 3.6 (1.4) | (110) | 3.3 (1.3) | (99) | 2.8 (1.5) |

**Appendix 2a: Distribution of the 11 PACIC items, by English-speaking countries (%)**

|  | **Item** | **Never** | **Generally not** | **Sometimes** | **Most of the time** | **Always** | **Missing values** |
| --- | --- | --- | --- | --- | --- | --- | --- |
| **USA** | **1** | 14.0 | 18.1 | 17.2 | 16.6 | 33.2 | 0.9 |
|  | **2** | 1.5 | 0.3 | 1.2 | 12.2 | 84.9 | 0.0 |
|  | **3** | 5.3 | 18.1 | 16.9 | 21.1 | 38.6 | 0.0 |
|  | **4** | 5.9 | 8.6 | 4.2 | 8.0 | 72.7 | 0.6 |
|  | **5** | 22.6 | 26.7 | 16.0 | 9.5 | 24.3 | 0.9 |
|  | **6** | 5.6 | 8.9 | 12.8 | 19.3 | 53.4 | 0.0 |
|  | **7** | 8.0 | 14.2 | 10.4 | 20.5 | 46.9 | 0.0 |
|  | **8** | 11.3 | 13.4 | 11.3 | 16.9 | 46.0 | 1.2 |
|  | **9** | 8.6 | 9.2 | 19.3 | 18.1 | 44.5 | 0.3 |
|  | **10** | 8.0 | 12.5 | 16.6 | 15.4 | 46.9 | 0.6 |
|  | **11** | 9.8 | 13.1 | 19.0 | 22.9 | 34.4 | 0.9 |
| **Canada** | **1** | 24.6 | 26.4 | 15.5 | 10.0 | 23.6 | 0.0 |
|  | **2** | 3.6 | 0.9 | 2.7 | 12.7 | 80.0 | 0.0 |
|  | **3** | 17.3 | 22.7 | 20.9 | 16.4 | 21.8 | 0.9 |
|  | **4** | 26.4 | 18.2 | 12.7 | 10.9 | 30.0 | 1.8 |
|  | **5** | 32.7 | 25.5 | 13.6 | 5.5 | 20.9 | 1.8 |
|  | **6** | 10.0 | 8.2 | 20.0 | 23.6 | 38.2 | 0.0 |
|  | **7** | 20.9 | 21.8 | 14.6 | 16.4 | 25.5 | 0.9 |
|  | **8** | 20.9 | 19.1 | 15.5 | 15.5 | 26.4 | 2.7 |
|  | **9** | 17.3 | 13.6 | 18.2 | 17.3 | 32.7 | 0.9 |
|  | **10** | 23.6 | 21.8 | 22.7 | 6.4 | 23.6 | 1.8 |
|  | **11** | 29.1 | 17.3 | 20.9 | 10.0 | 21.8 | 0.9 |

*Items: 1) Given choices about treatment to think about; 2) Satisfied that my care was well organized*

*3) Helped to set specific goals to improve my eating or exercise; 4) Given a copy of my treatment plan; 5) Encouraged to go to a specific group or class to help me cope with my heart transplantation; 6) Asked questions, either directly or with a questionnaire, about my health habits; 7) Helped to make a treatment plan that I could carry out in my daily life; 8) Helped to plan ahead so I could take care of my transplanted heart even in hard times; 9) Asked how my heart transplantation affects my life; 10) Contacted after a visit to see how things were going; 11) Told how my visits with other types of doctors, like an eye doctor or surgeon, helped my treatment (9).*

**Appendix 2b: Distribution of the 11 PACIC items, by English-speaking countries (%)**

|  | **Item** | **Never** | **Generally not** | **Sometimes** | **Most of the time** | **Always** | **Missing values** |
| --- | --- | --- | --- | --- | --- | --- | --- |
| **UK** | **1** | 25.5 | 16.3 | 17.4 | 18.4 | 20.4 | 2.0 |
|  | **2** | 0.0 | 0.0 | 0.0 | 10.2 | 88.8 | 1.0 |
|  | **3** | 25.5 | 13.3 | 23.5 | 17.4 | 19.4 | 1.0 |
|  | **4** | 33.7 | 15.3 | 7.1 | 11.2 | 30.6 | 2.0 |
|  | **5** | 53.1 | 20.4 | 8.2 | 8.2 | 9.2 | 1.0 |
|  | **6** | 18.4 | 8.2 | 26.5 | 17.4 | 29.6 | 0.0 |
|  | **7** | 40.8 | 16.3 | 12.2 | 9.2 | 19.4 | 2.0 |
|  | **8** | 35.7 | 13.3 | 13.3 | 13.3 | 22.5 | 2.0 |
|  | **9** | 16.3 | 15.3 | 18.4 | 16.3 | 30.6 | 3.1 |
|  | **10** | 34.7 | 22.5 | 14.3 | 8.2 | 19.4 | 1.0 |
|  | **11** | 40.8 | 21.4 | 9.2 | 9.2 | 18.4 | 1.0 |
| **Australia** | **1** | 19.6 | 19.6 | 23.5 | 17.7 | 19.6 | 0.0 |
|  | **2** | 0.0 | 0.0 | 3.9 | 17.7 | 78.4 | 0.0 |
|  | **3** | 19.6 | 19.6 | 25.5 | 9.8 | 25.5 | 0.0 |
|  | **4** | 27.5 | 21.6 | 9.8 | 3.9 | 35.3 | 2.0 |
|  | **5** | 41.2 | 9.8 | 15.7 | 7.8 | 25.5 | 0.0 |
|  | **6** | 23.5 | 17.7 | 19.6 | 17.7 | 21.6 | 0.0 |
|  | **7** | 31.4 | 11.8 | 23.5 | 11.8 | 21.6 | 0.0 |
|  | **8** | 25.5 | 17.7 | 11.8 | 19.6 | 25.5 | 0.0 |
|  | **9** | 17.7 | 15.7 | 17.7 | 23.5 | 25.5 | 0.0 |
|  | **10** | 25.5 | 21.6 | 31.4 | 5.9 | 15.7 | 0.0 |
|  | **11** | 17.7 | 23.5 | 25.5 | 11.8 | 21.6 | 0.0 |

*Items: 1) Given choices about treatment to think about; 2) Satisfied that my care was well organized*

*3) Helped to set specific goals to improve my eating or exercise; 4) Given a copy of my treatment plan; 5) Encouraged to go to a specific group or class to help me cope with my heart transplantation; 6) Asked questions, either directly or with a questionnaire, about my health habits; 7) Helped to make a treatment plan that I could carry out in my daily life; 8) Helped to plan ahead so I could take care of my transplanted heart even in hard times; 9) Asked how my heart transplantation affects my life; 10) Contacted after a visit to see how things were going; 11) Told how my visits with other types of doctors, like an eye doctor or surgeon, helped my treatment (9).*

**Appendix 3a: Distribution of the 11 PACIC items by language groups (%)**

|  | **Item** | **Never** | **Generally not** | **Sometimes** | **Most of the time** | **Always** | **Missing values** |
| --- | --- | --- | --- | --- | --- | --- | --- |
| **French** | **1** | 27.5 | 10.1 | 17.5 | 11.6 | 30.2 | 3.2 |
|  | **2** | 2.7 | 0.5 | 3.7 | 22.8 | 69.8 | 0.5 |
|  | **3** | 13.2 | 9.5 | 21.7 | 26.5 | 25.4 | 3.7 |
|  | **4** | 20.6 | 3.2 | 5.3 | 9.0 | 56.6 | 5.3 |
|  | **5** | 47.6 | 8.5 | 16.9 | 8.5 | 14.3 | 4.2 |
|  | **6** | 13.8 | 9.0 | 24.9 | 24.9 | 23.8 | 3.7 |
|  | **7** | 30.2 | 6.4 | 15.9 | 14.3 | 28.0 | 5.3 |
|  | **8** | 25.9 | 7.4 | 14.3 | 16.4 | 30.7 | 5.3 |
|  | **9** | 19.6 | 9.5 | 21.7 | 18.5 | 28.0 | 2.7 |
|  | **10** | 31.8 | 11.6 | 23.8 | 12.2 | 16.9 | 3.7 |
|  | **11** | 22.2 | 10.1 | 15.9 | 25.9 | 22.8 | 3.2 |
| **German** | **1** | 17.0 | 21.7 | 15.1 | 21.7 | 23.6 | 0.9 |
|  | **2** | 0.9 | 0.9 | 2.8 | 21.7 | 73.6 | 0.0 |
|  | **3** | 9.4 | 13.2 | 15.1 | 23.6 | 37.7 | 0.9 |
|  | **4** | 11.3 | 5.7 | 2.8 | 9.4 | 70.8 | 0.0 |
|  | **5** | 30.2 | 25.5 | 12.3 | 16.0 | 14.2 | 1.9 |
|  | **6** | 10.4 | 15.1 | 23.6 | 15.1 | 34.0 | 1.9 |
|  | **7** | 19.8 | 30.2 | 6.6 | 14.2 | 27.4 | 1.9 |
|  | **8** | 15.1 | 22.6 | 8.5 | 15.1 | 37.7 | 0.9 |
|  | **9** | 8.5 | 12.3 | 16.0 | 23.6 | 38.7 | 0.9 |
|  | **10** | 20.8 | 18.9 | 16.0 | 21.7 | 22.6 | 0.0 |
|  | **11** | 8.5 | 15.1 | 16.0 | 23.6 | 34.9 | 1.9 |
| **Dutch** | **1** | 29.4 | 17.7 | 15.7 | 21.6 | 13.7 | 2.0 |
|  | **2** | 7.8 | 2.0 | 2.0 | 33.3 | 54.9 | 0.0 |
|  | **3** | 25.5 | 7.8 | 15.7 | 27.5 | 23.5 | 0.0 |
|  | **4** | 33.3 | 11.8 | 13.7 | 13.7 | 27.5 | 0.0 |
|  | **5** | 41.2 | 9.8 | 23.5 | 19.6 | 5.9 | 0.0 |
|  | **6** | 9.8 | 2.0 | 13.7 | 31.4 | 43.1 | 0.0 |
|  | **7** | 29.4 | 2.0 | 13.7 | 25.5 | 27.5 | 2.0 |
|  | **8** | 33.3 | 9.8 | 11.8 | 21.6 | 23.5 | 0.0 |
|  | **9** | 21.6 | 3.9 | 23.5 | 21.6 | 29.4 | 0.0 |
|  | **10** | 45.1 | 9.8 | 11.8 | 9.8 | 23.5 | 0.0 |
|  | **11** | 27.5 | 9.8 | 23.5 | 25.5 | 13.7 | 0.0 |

*Items: 1) Given choices about treatment to think about; 2) Satisfied that my care was well organized*

*3) Helped to set specific goals to improve my eating or exercise; 4) Given a copy of my treatment plan; 5) Encouraged to go to a specific group or class to help me cope with my heart transplantation; 6) Asked questions, either directly or with a questionnaire, about my health habits; 7) Helped to make a treatment plan that I could carry out in my daily life; 8) Helped to plan ahead so I could take care of my transplanted heart even in hard times; 9) Asked how my heart transplantation affects my life; 10) Contacted after a visit to see how things were going; 11) Told how my visits with other types of doctors, like an eye doctor or surgeon, helped my treatment (9).*

**Appendix 3b: Distribution of the 11 PACIC items by language groups (%)**

|  | **Item** | **Never** | **Generally not** | **Sometimes** | **Most of the time** | **Always** | **Missing values** |
| --- | --- | --- | --- | --- | --- | --- | --- |
| **Spanish** | **1** | 68.6 | 4.5 | 5.4 | 3.6 | 14.4 | 3.6 |
|  | **2** | 0.9 | 0.5 | 1.4 | 10.8 | 86.1 | 0.5 |
|  | **3** | 9.0 | 3.1 | 12.6 | 17.0 | 57.0 | 1.4 |
|  | **4** | 0.9 | 0.0 | 0.9 | 10.3 | 87.0 | 0.9 |
|  | **5** | 41.3 | 5.4 | 13.0 | 10.8 | 26.5 | 3.1 |
|  | **6** | 45.3 | 8.5 | 13.0 | 6.7 | 22.9 | 3.6 |
|  | **7** | 13.5 | 0.9 | 5.8 | 14.4 | 64.6 | 0.9 |
|  | **8** | 17.5 | 2.7 | 7.2 | 16.1 | 55.6 | 0.9 |
|  | **9** | 10.3 | 2.2 | 10.8 | 15.3 | 60.1 | 1.4 |
|  | **10** | 22.9 | 9.9 | 10.3 | 7.6 | 48.0 | 1.4 |
|  | **11** | 26.0 | 5.8 | 11.7 | 9.0 | 46.2 | 1.4 |
| **Italian** | **1** | 29.0 | 23.7 | 26.3 | 2.6 | 11.4 | 7.0 |
|  | **2** | 0.9 | 0.0 | 0.0 | 11.4 | 87.7 | 0.0 |
|  | **3** | 7.0 | 7.9 | 12.3 | 21.1 | 50.9 | 0.9 |
|  | **4** | 0.0 | 0.9 | 2.6 | 8.8 | 86.8 | 0.9 |
|  | **5** | 29.0 | 26.3 | 15.8 | 5.3 | 21.9 | 1.8 |
|  | **6** | 6.1 | 4.4 | 19.3 | 14.0 | 55.3 | 0.9 |
|  | **7** | 0.9 | 2.6 | 7.0 | 14.0 | 75.4 | 0.0 |
|  | **8** | 11.4 | 13.2 | 19.3 | 12.3 | 43.0 | 0.9 |
|  | **9** | 9.7 | 15.8 | 27.2 | 14.0 | 32.5 | 0.9 |
|  | **10** | 6.1 | 13.2 | 28.1 | 14.9 | 36.8 | 0.9 |
|  | **11** | 17.5 | 21.9 | 22.8 | 8.8 | 29.0 | 0.0 |
| **Portuguese** | **1** | 35.4 | 7.1 | 7.1 | 11.1 | 35.4 | 4.0 |
|  | **2** | 3.0 | 1.0 | 1.0 | 5.1 | 89.9 | 0.0 |
|  | **3** | 9.1 | 5.1 | 7.1 | 12.1 | 66.7 | 0.0 |
|  | **4** | 25.3 | 3.0 | 4.0 | 12.1 | 52.5 | 3.0 |
|  | **5** | 39.4 | 13.1 | 9.1 | 12.1 | 25.3 | 1.0 |
|  | **6** | 28.3 | 7.1 | 21.2 | 10.1 | 32.3 | 1.0 |
|  | **7** | 35.4 | 13.1 | 9.1 | 5.1 | 36.4 | 1.0 |
|  | **8** | 24.2 | 4.0 | 3.0 | 15.2 | 52.5 | 1.0 |
|  | **9** | 30.3 | 9.1 | 10.1 | 14.1 | 32.3 | 4.0 |
|  | **10** | 28.3 | 9.1 | 12.1 | 18.2 | 30.3 | 2.0 |
|  | **11** | 18.2 | 9.1 | 14.1 | 15.2 | 41.4 | 2.0 |

*Items: 1) Given choices about treatment to think about; 2) Satisfied that my care was well organized*

*3) Helped to set specific goals to improve my eating or exercise; 4) Given a copy of my treatment plan; 5) Encouraged to go to a specific group or class to help me cope with my heart transplantation; 6) Asked questions, either directly or with a questionnaire, about my health habits; 7) Helped to make a treatment plan that I could carry out in my daily life; 8) Helped to plan ahead so I could take care of my transplanted heart even in hard times; 9) Asked how my heart transplantation affects my life; 10) Contacted after a visit to see how things were going; 11) Told how my visits with other types of doctors, like an eye doctor or surgeon, helped my treatment (9).*

| **Appendix 4: relations between PACIC global score and other variables by English speaking countries** | | | | | | | | | | |
| --- | --- | --- | --- | --- | --- | --- | --- | --- | --- | --- |
|  | English (n=596) | | USA (n=337) | | CANADA (n=110) | | UK (n=98) | | AUSTRALIA (n=51) | |
| Advice to exercise (yes=1, no=0) (1) | 1.0 | *** | 0.9 | ** | 0.9 | *** | 0.3 | ns | 0.7 | * |
| Transplant team discussed intake immunosuppressants (yes=1, no=0) (1) | 1.1 | *** | 0.8 | *** | 0.8 | * | 1.2 | *** |  |  |
| Satisfaction with transplant team (2) | 0.364 | *** | 0.353 | *** | 0.235 | * | 0.405 | *** | 0.369 | ** |
| Trust in transplant team (2) | 0.268 | *** | 0.311 | *** | 0.18 | . | 0.147 | ns | 0.32 | * |
| *Statistical test: (1) t-test: mean differences, (2) spearman correlation; p-value: *** p<0.001; ** p<0.01; * p<0.05; p<0.10; ns non-significant p-value; N.B.: empty cells when category "no" less than 10 persons.* | | | | | | | | | | |
